# A LN/Si-Based SAW Pressure Sensor

**DOI:** 10.3390/s18103482

**Published:** 2018-10-16

**Authors:** Pascal Nicolay, Hugo Chambon, Gudrun Bruckner, Christian Gruber, Sylvain Ballandras, Emilie Courjon, Matthias Stadler

**Affiliations:** 1 Carinthian Tech Research (CTR AG), Europastrasse 12, 9524 Villach, Austria; hugo.chambon@ctr.at (H.C.); gudrun.bruckner@ctr.at (G.B.); christian.gruber@ctr.at (C.G.); 2 frec|n|sys, TEMIS Innovation, 18 Rue Alain Savary, 25000 Besançon, France; sylvain.ballandras@frecnsys.fr (S.B.); emilie.courjon@frecnsys.fr (E.C.); 3 UNISENSOR AG, Bahnstrasse 12A, 8544 Attikon, Switzerland; m.stadler@unisensor.ch

**Keywords:** SAW pressure sensor, multilayer substrate, thin LN membranes, LN/Si, wafer-bonding

## Abstract

Surface Acoustic Wave (SAW) sensors are small, passive and wireless devices. We present here the latest results obtained in a project aimed at developing a SAW-based implantable pressure sensor, equipped with a well-defined, 30 μm-thick, 4.7 mm-in-diameter, Lithium Niobate (LN) membrane. A novel fabrication process was used to solve the issue of accurate membrane etching in LN. LN/Si wafers were fabricated first, using wafer-bonding techniques. Grinding/polishing operations followed, to reduce the LN thickness to 30 μm. 2.45 GHz SAW Reflective Delay-Lines (R-DL) were then deposited on LN, using a combination of e-beam and optical lithography. The R-DL was designed in such a way as to allow for easy temperature compensation. Eventually, the membranes were etched in Si. A dedicated set-up was implemented, to characterize the sensors versus pressure and temperature. The achieved pressure accuracy is satisfactory (±0.56 mbar). However, discontinuities in the response curve and residual temperature sensitivity were observed. Further experiments, modeling and simulations were used to analyze the observed phenomena. They were shown to arise essentially from the presence of growing thermo-mechanical strain and stress fields, generated in the bimorph-like LN/Si structure, when the temperature changes. In particular, buckling effects explain the discontinuities, observed around 43 °C, in the response curves. Possible solutions are suggested and discussed.

## 1. Introduction

Surface Acoustic Wave (SAW) sensors are small, robust, passive and wireless devices that do not require any embedded electronics [1]. This presents numerous advantages, for a wide range of applications [2,3,4,5]. They are, in particular, well-suited for applications in extreme environment [6,7,8]. SAW sensors also have great potential for Structural Health Monitoring and Smart Materials applications, as they can be implanted in various kinds of parts and structures [9]. SAW sensors are also well-suited for applications where implanted, maintenance-free bio-sensors are required [10,11,12,13]. Membrane-based SAW pressure sensors are, for instance, a promising solution for the in situ measurement of intracranial pressure (ICP). The proof-of-concept of an implantable SAW intracranial pressure sensor was demonstrated in 2013 by Bruckner et al. [14]. Here, a demonstrator based on a 2.45 GHz SAW Reflective Delay Line (R-DL) deposited on Y-Z Lithium Niobate (LNYZ) was used. It responded linearly to applied pressure around 1 bar, with a pressure sensitivity of 5 rad/bar. Still, it proved very difficult to fabricate well-defined thin membranes in Lithium Niobate. Different drilling techniques, from laser to ultrasound, were tried unsuccessfully (unpublished work). A new solution was needed, to overcome these limitations.

In this paper, we present the results of a complete study aimed at developing a significantly improved 2.45 GHz SAW R-DL pressure sensor, equipped with a well-defined, 4.7 mm-in-diameter, 30 μm-thick Lithium Niobate membrane. A novel fabrication procedure was used, to solve the membrane etching issues. Namely, a LN/Si composite wafer was used instead of a pure LN one. The process flow was analogous to the one used in the past by Grousset et al., to fabricate Quartz-based SAW pressure sensors [15]. Here, LN-based R-DL(s) were used instead of Quartz-based resonators, as some of our targeted applications require multiple-sensor interrogation and Radio-Frequency IDentification (RFID) capabilities. R-DL signals can indeed overlap easier than resonator signals, and R-DL designs can easily be upgraded to include series of identification peaks [16]. However, the use of the composite LN/Si wafers resulted in a series of fabrication issues, which were eventually solved through the use of a combination of e-beam and optical lithography. The fabrication process, issues and solutions are described in Section 2.1.

An improved ‘temperature-compensated’ R-DL Design was also developed, and implemented. It is described in Section 2.2. Small connection pads were used, to reduce capacitive coupling between the two polarities of the R-DL’s Inter-Digital Transducer (IDT). Indeed, large pads were found to be strongly electrically coupled, due the presence of a bonding Au/Cr layer at the LN/Si interface. This generated electrical matching issues. It also generated extra losses, through Bulk Acoustic waves (BAW) excitation in the Si wafer carrier.

A test set-up was designed and implemented, to characterize the sensors in pressure and temperature. The set-up and measurement procedure are described in Section 2.3.

Results are presented in Section 3. The larger membrane (4.7 mm versus 2 mm in Reference [14]) made it possible to achieve a final pressure accuracy of ±0.56 mbar, in a 1–1.03 bar pressure range. The observed pressure sensitivity was satisfactory, but the characterization results showed two critical issues: the sensors were still sensitive to temperature (at constant pressure), and discontinuities were observed in the response curve (measured at constant pressure), around 43 °C.

Further experiments, modeling and simulations were performed to analyze and understand the observed phenomena. They were shown to arise essentially from growing thermo-mechanical strain and stress fields, generated in the bimorph-like LN/Si structure, when submitted to temperature changes. In particular, buckling effects were found to be responsible for the observed discontinuities, in the response curves. Detailed explanations are provided in Section 4. Possible solutions are discussed before the conclusion.

## 2. Materials and Methods

### 2.1. Fabrication Process

**Figure 1:**
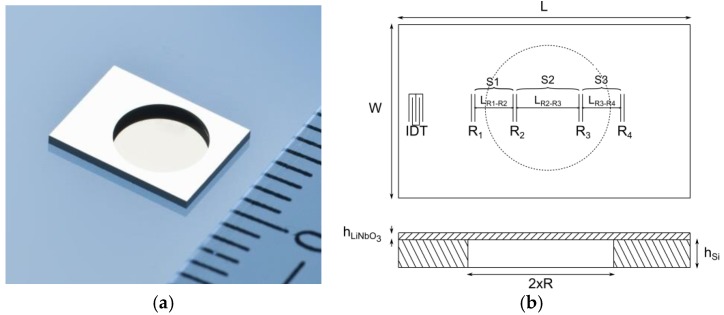
Membrane-based LN/Si Surface Acoustic Wave (SAW) pressure sensor: (**a**) pictured from below. The Inter Digital Transducer (IDT) and reflectors are located on the other side of the element. The membrane is 30 μm-thick. Its diameter is 4.7 mm; (**b**) R-DL and sensor design (schematics).

The membranes were fabricated as follows. A thick LN wafer was first bonded to a 350 μm-thick Si carrier wafer, using a 300 nm-thick Au/Cr bonding layer. The LN wafer was then thinned down to the required thickness of 30 μm, before the surface was polished and the IDT and reflectors were processed. Finally, the membranes were dry-etched in the Si wafer, using standard Bosch process. High-quality, 4.7 mm-in-diameter membranes were obtained (see Figure 1a).

An R-DL mainly comprises one IDT and a series of reflectors, located a few millimeters away from the IDT. The IDT is used to convert electrical signals into acoustic waves, and vice versa. The reflectors reflect fractions of the wave energy, back to the IDT. Consequently, the impulse response of an R-DL shows well-defined peaks, in time domain [17]. The position of a peak is, among others, temperature and strain dependent. This is what makes it possible to use R-DL SAW sensors for pressure and temperature measurement purposes. 

In our case, the R-DL design was analogous to the one presented in Figure 1b, with two reflectors (R2, R3) located on the membrane, and two reflectors (R1, R4) located outside it. Its fabrication proved, however, to be quite a challenging task. The IDT and reflectors are thin metal gratings. In our Design (i.e., SAW R-DL operating at 2.45 GHz), the Ti/Al fingers had a thickness of ~60 nm and a pitch of ~0.7 μm. The metallization ratio was 50% (i.e., the finger width and the gap between two consecutive fingers were both equal to ~0.35 μm), in both the IDT and the reflectors.

High quality gratings are routinely fabricated on LN substrates [18]. However, in the case of LN/Si, the presence of a thin reflective Au/Cr bonding layer between the LN and the Si wafers, as well as the slight curvature of the wafer over the propagation path (which is longer than 5 mm, in our case) strongly complicates the photolithography steps. The exposure is perturbed by the light diffused and reflected at the Au/Cr interface, while the focus is to be constantly adjusted to compensate for the wafer curvature, from one chip to the other. In addition, the curvature can be large enough to generate focus issues on one single chip, as the IDT is located several millimeters away from the fourth reflector. The initial curvature of the carrier Si wafer also generates thickness variations of the LN layer, at the end of the thinning process. This generates additional exposure issues, from chip to chip. The thickness variation on one LN/Si wafer was characterized experimentally. It was found to vary from 27 μm to 38 μm, over the wafer surface. 

**Figure 2:**
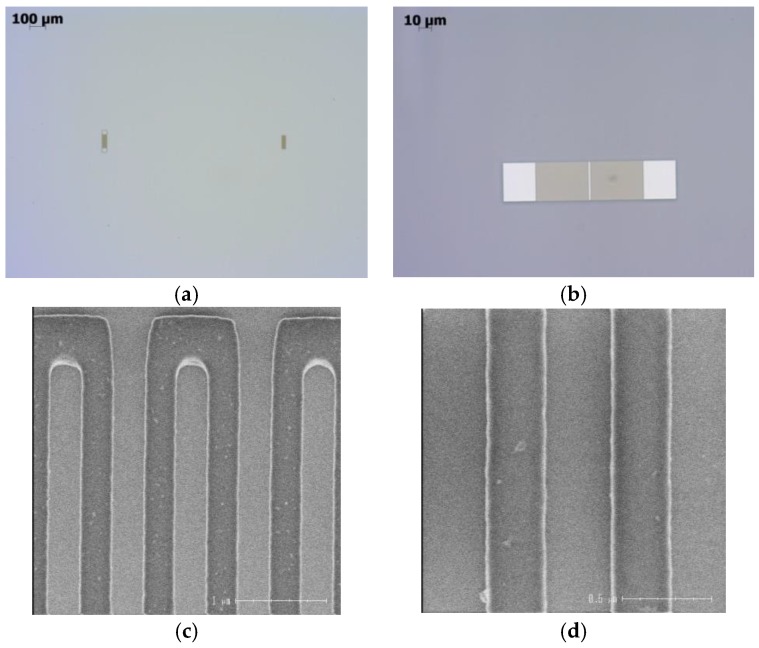
IDT and reflector R1, after first front-end manufacturing sequence: (**a**) large view of the IDT and reflector R1(optical microscopy); (**b**) closer view of the IDT. Only the fingers and bus bars were manufactured, at this stage; (**c**) Critical Dimension-Scanning Electron Microscopy (CDSM) view of the IDT, after lift-off; (**d**) closer CDSM view of the IDT fingers.

To solve these fabrication issues, a combination of e-beam and optical lithography was employed. E-beam lithography was used to pattern the fingers (i.e., all fine structures), whereas optical lithography was used to pattern the bus bars and connection pads (i.e., coarse structures). In addition, simple open reflectors were used instead of shorted ones to simplify their layout, as no bus bars are needed in this case. Post-processing control of the lithography quality was performed over the whole wafer, using optical microscopy and Critical Dimension-Scanning Electron Microscopy (CDSEM) techniques. Most of the devices were correctly patterned. A systematic deviation of the metal ratio was observed. The fingers were slightly larger than expected, with an observed width of 360–370 nm. A large, 90°-rotated view of the IDT is shown in Figure 2b. The IDT is actually made of two identical IDTs connected in series or rather, of one wide IDT split in two using a central bus bar. A closer inspection showed some shift (~50 nm) between the upper half and the lower half of the IDT. This shift, which is too small to be seen on the figure, resulted from alignment issues during e-beam writing (the e-beam writing sequence imposed the upper half to be written before the lower half). To use a plain IDT instead of a split one would easily solve the latter issue, at the expense of a slight degradation of the IDT performances. SEM pictures of the fabricated IDT and reflectors are presented in Figure 2. 

Special attention must also be paid to the number of fingers used in each reflector. To enable efficient post-processing of the sensor’s response, the four peaks must be of equal or similar amplitude. To fulfill this requirement, the first reflector (R1) must contain a few fingers only, while the fourth reflector (R4) must contain more than twenty. Indeed, due to propagation losses, R4 sees much less incoming energy than R1, as it is located much further away from the IDT. Moreover, some appreciable fraction of the energy is already reflected back by R1, R2 and R3. In our case, three fingers only were used for R1. This was an ill-fated decision, as lithography imperfections resulted in at least one missing finger per reflector. As a result, the R1 structures did not work anymore, and the R1 peaks were absent from the sensor signatures (see Section 3). It is therefore recommended to use at least five fingers for R1. More advanced grating structures could also be used, to improve the reflectivity of the reflectors [19].

**Figure 3:**
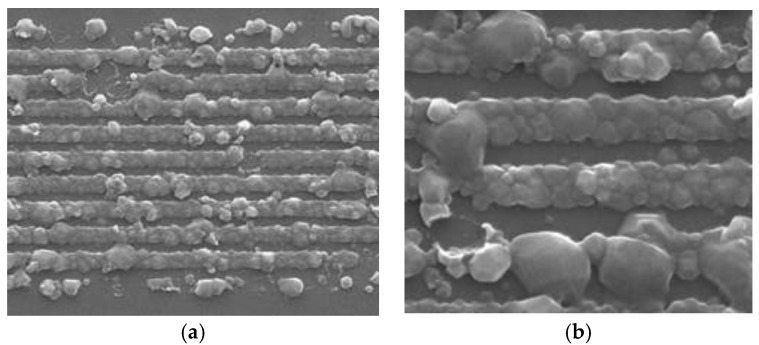
(**a**) SEM picture of a reflector (located on the membrane), after membrane etching and improper IDT and reflector protection; (**b**) zoom on a few fingers.

Finally, it is of paramount importance to protect the IDT and reflectors during membrane etching. SEM pictures of a reflector (located on the membrane) after improper membrane etching are shown in Figure 3. In that case, the almost complete destruction of the fingers was plausibly due to the action of Fluorine (Bosch process), whose presence was afterwards confirmed by Energy-dispersive X-ray spectroscopy (EDX). Electrode degradation on LiNbO3 substrates was also reported by several authors [20], in the absence of any (DRIE) etching process. These authors concluded that the origin of the defects was related to electro-migration. Even if usual SAW R-DL sensors are hardly submitted to incident electrical power-levels larger than 10 dBm, the Bosch process might help activate the phenomenon and lead to the early degradation of the IDT and reflectors. It would therefore be wise to passivate the electrodes in any case, to definitely get rid of such degradation.

### 2.2. Sensor Design

Our Design is similar to the one presented in Reference [14], and is an evolution of the one described in Reference [21]. The reflectors divide the propagation path into three well-defined sections, S1, S2 and S3 (see Figure 1b). The sections S1 (L_R1−R2_) and S3 (L_R3−R4_) are of equal length (L_R1−R2_ = L_R3−R4_ = 1.572 mm, center-to-center). The section S2 (L_R2−R3_) is twice longer. R1 is located 1.197 mm away from the IDT (center-to-center). In an R-DL, the temperature sensitivity is directly proportional to the propagation length and thus, to the echo time (τ) or to the phase difference between the excitation signal and the reflected one (ϕ). The quantity S = ϕ(S2) − [ϕ(S1) + ϕ(S3)] is therefore temperature independent. Here, ϕ(S1), ϕ(S2) and ϕ(S3) refer to the phase difference between the peaks R2 and R1, R3 and R2, and R4 and R3, respectively. In addition, R2 and R3 are positioned on the membrane, at the locations where the longitudinal strain is always zero, whatever the applied hydrostatic pressure (in our case, the respective positions of R2 and R3 were determined through preliminary Finite Element Modeling (FEM) simulations). Consequently, the average longitudinal strain in Section 1 and Section 3 (on the first hand), and in Section 2 (on the other hand) are always of opposite sign, and so are the respective strain sensitivities of the quantities ϕ(S2) and [ϕ(S1) + ϕ(S3)]. As a result, the quantity S shall be temperature insensitive but highly pressure dependent.

**Figure 4:**
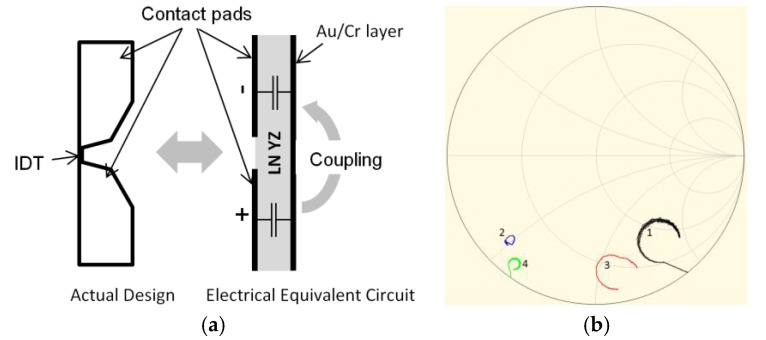
(**a**) Capacitive coupling issue between two connection pads of an IDT (red circles), in the presence of an Au/Cr bonding layer; (**b**) Effect, in the Smith chart, of the Pad-to-Pad capacitive coupling on the IDT impedance. Black (1): reference IDT, mounted on pure LN (no capacitive coupling); Blue (2): IDT with large pads; Red (3): IDT after reduction of the pads dimension, using Laser ablation; Green (4): calculated response of the reference IDT (black curve), shunted with two series 3.95 pF capacitors.

The presence of the Au/Cr bonding layer at the LN/Si interface generates two main electrical issues. The first one is direct capacitive coupling between the two connection pads of the IDT (see Figure 4a). This can significantly impact the electrical behavior of the R-DL, by strongly reducing its reactance. In Figure 4b, the impedance characteristics of the same IDT with different pad configurations are shown, for comparison purposes. A reference IDT was tested first (black curve). It was deposited on a pure LN substrate and equipped with the large connection pads shown in Figure 4a. As there was no Au/Cr layer here, no capacitive coupling could occur. The response of an identical IDT deposited on LN/{Au/Cr}/Si and equipped with the same large pads is shown in blue. The response is strongly degraded, and shifted towards the low-reactance region of the Smith chart (blue curve). Laser ablation was then used to reduce the size of the pads, by almost 50%. The red curve shows the response of the IDT on LN/{Au/Cr}/Si, after pad-size reduction. It is much closer to the reference one. The green curve was obtained using a circuit simulator. The objective was to reproduce the capacitive-coupling effect of the large pads. Their capacitance was approximated first to 3.95 pF, using only the surface of the pads, and the thickness and dielectric coefficient ε_33_ of LNYZ. Two series 3.95 pF capacitors were then used to shunt the reference IDT, in the simulated circuit. The green curve is close to the blue one, which confirms that the signal degradation is due to the use of large pads that shunt the IDT through capacitive coupling, in the presence of the Au/Cr bonding layer.

**Figure 5:**
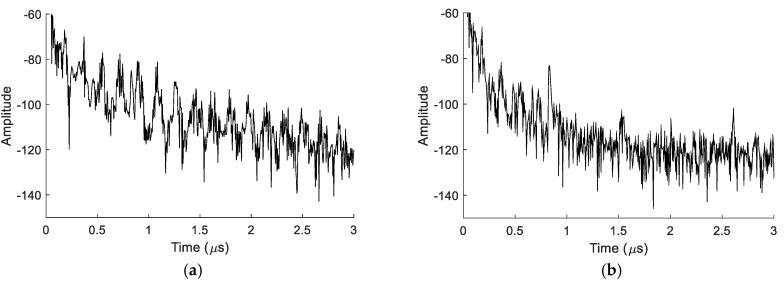
Improvement of the impulse response, using smaller pads and an absorbing layer (nail polish) on the back side, to reduce the amplitude of spurious BAW noise: (**a**) Before; (**b**) After.

A second issue arises from the use of the Au/Cr layer. As LN is piezoelectric, the Pad/LN/{Au/Cr} capacitors also behave as electro-mechanical resonators, or Bulk Acoustic Wave resonators (BAW). In our case, the BAW resonators are solidly mounted on a Si substrate, into which they can excite travelling BAWs. These waves travel back and forth between the bottom and top surfaces of the Si wafer. This generates additional periodic echoes in the sensor response (i.e., spurious peaks). The effect can be compared to high overtone resonator structures yielding a spectral/time domain comb corresponding to all possible bulk resonances in the composite plate. In Figure 5a, the time response of a selected device from an earlier generation is shown. Here, the IDT was equipped with large pads, to facilitate wafer-prober measurements. The useful signals (i.e., the peaks) are buried in spurious BAW noise. In Figure 5b, the time response of the same device is shown, after laser ablation of the pads (to strongly reduce their surface) and application of nail polish on the back side, to attenuate the emitted BAW. The amplitude of the spurious BAW noise is strongly reduced, which makes some of the useful peaks detectable again. This proves that smaller pads and viscous damping (on the back side) are efficient solutions to attenuate the spurious BAW peaks. However, there is no obvious solution to further reduce or (ideally) fully suppress the emission of BAW in Si, as they are a direct consequence of the utilization of a conductive Au/Cr bonding layer.

It is therefore important to reduce the size of the pads to the minimum possible. It is also important to position the pads close to the IDT bus bars, in order to reduce the size of the thin connection lines between the bus bars and the pads. Indeed, due to their own parasitic inductance, long lines can degrade the signal again, thus canceling the benefits of using smaller pads. We eventually used 150 μm × 200 μm pads, separated by a gap of 1260 μm. We used thin connection lines, (width < 75 μm), to connect the pads to the IDT bus bars.

### 2.3. Characterization Set-Up and Procedure

**Figure 6:**
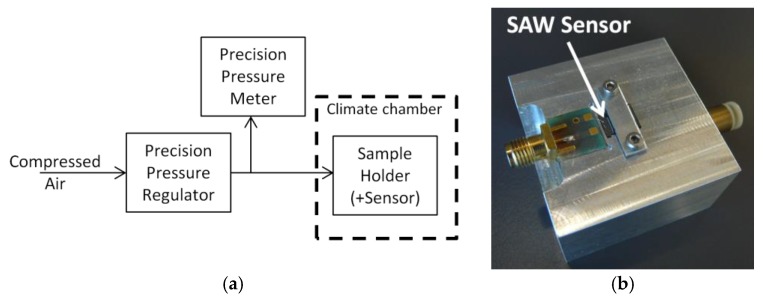
Test Set-Up: (**a**) Set-Up schematics; (**b**) Sample holder.

The schematics of the test-bench we used to characterize the sensor are shown in Figure 6a. First, a precision pressure regulator (IMI Norgren 11-818-987, 2355 Wiener Neudorf, Austria) was used to reduce the input pressure from 7 bars (compressed air) to the required applied pressure, in the range 1–1.03 bar. The pressure was measured in parallel, using a Bronkhorst digital precision pressure meter (EL-PRESS). The tuned pressure was then applied on the membrane, using a dedicated sample holder (see Figure 6b). The sample holder comprised a pressure pipe (located inside the jig), a cavity where to place the sensor, and a PCB with its SMA (SubMiniature version A) connector. The sensor’s connection pads were connected to the Printed Circuit Board (PCB) using standard gold bond wires. A proprietary SAW reader [22] was used to read the sensor (set in phase-tracking mode) and follow the evolution of the phase of the reflection peaks, versus applied pressure (at constant temperature) or applied temperature (at constant pressure).

## 3. Results

**Figure 7:**
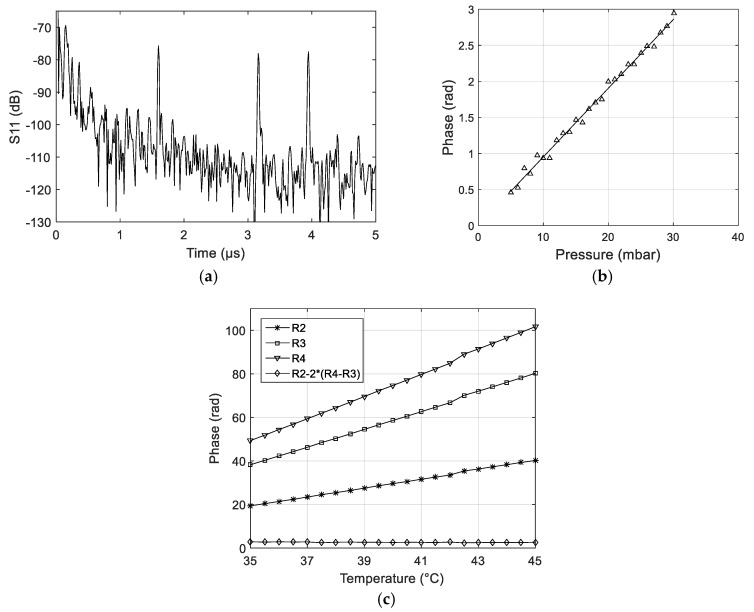
(**a**) LN/Si SAW R-DL response, in time domain; (**b**) Phase-shift vs. applied pressure; (**c**) Phase-shift vs. temperature (no applied pressure). The residual temperature sensitivity (diamond line) is close to zero.

The response in time domain of one selected LN/Si SAW pressure sensor is shown in Figure 7a. As mentioned in Section 2.1, improper design of R1 (only three fingers) made it highly sensitive to lithography issues. In that particular case, slight damages to R1 were enough to make it non-functional. However, as Section S1 and S3 are identical (see Figure 1b), it was still possible to perform the measurements, using peaks R2, R3 and R4 only. In this case, the data-processing algorithm was asked to compute the quantity S = ϕ(S2) − [2 × ϕ(S3)] or S = ϕ(R2) − [2 × ϕ(R4 − R3)] instead of S = ϕ(S2) − [ϕ(S1) + ϕ(S3)]. The pressure sensitivity at constant temperature (+25 °C) and the residual temperature sensitivity (i.e., at constant, atmospheric pressure) were characterized. The results are presented in Figure 7b,c.

The slope of the linear fit in Figure 7b determines the sensitivity of the sensor. It is 0.088 rad/mBar or 11.35 mBar/rad. According to the reading accuracy of CTR SAW Readers (±0.05 rad) [23], this corresponds to a pressure accuracy of ±0.56 mBar.

As we used a SAW reader to characterize the sensor, this accuracy is close to the one that we would obtain in wireless interrogation mode (here, a coaxial cable was used to connect the sample holder’s PCB to the SAW reader, instead of antennas).

The residual temperature sensitivity is close to zero. However, other sensors from the same fabrication run were shown to have non-zero residual temperature sensitivity. The residual sensitivity always stayed below 1 degree of phase in the 35–45 °C range, but this value is already large enough to prevent the sensors from being used, as temperature-compensated pressure sensors. A second issue was observed, for most of the tested sensors, namely, a discontinuity in the Phase vs. Temperature curve, around 42–43 °C (see Figure 7c).

## 4. Discussion

### 4.1. Residual Temperature Sensitivity

The thermal coefficients of expansion (TCE) of LN and Si are different. They are respectively 7.5 ppm/K and 2.6 ppm/K, for LNYZ and Si, in the propagation direction. This generates thermally induced stress and strain fields in the structure, when the temperature changes.

Strain and stress fields affect the wave propagation properties of SAW devices. As mentioned already, that is (of course) why they can be used as strain and pressure sensors. That is also why growing thermo-mechanical strain fields result in residual temperature sensitivity, for the LN/Si sensors. This effect was accurately computed and accounted for, in previous work [24]. However, the temperature sensitivity of our sensor, which should be zero by design, is also influenced by any imperfection in the mounting of the device in its housing and by residual, unpredictable stress and strain fields after wafer-bonding. Consequently, it appears almost impossible to solve this issue other than by directly measuring the temperature and correcting the pressure readings accordingly.

A first, straightforward solution consists in measuring the temperature using an additional temperature sensor. To accurately measure the temperature at the pressure sensor location, both sensors need to be as close as possible to each other. A second R-DL could be used, next to the first one, to measure temperature only. However, such a solution would be suboptimal as it would require additional space and, more important, would reduce the reading range of the system. Indeed, the energy provided by the reader would be shared between the two R-DLs, which are connected to the same antenna.

Another, more elegant solution, would consist in extracting the temperature information from the existing design. As mentioned and used above, the combination of the properties of Sections S1, S2 and S3 provides temperature-compensated pressure readings, using S = ϕ(S2) − [ϕ(S1) + ϕ(S3)]. On the other hand, the quantity U = ϕ(S2) + [ϕ(S1) + ϕ(S3)] would provide temperature readings, however with residual pressure sensitivity (the pressure sensitivity of S1 + S3 is not exactly opposite to the pressure sensitivity of S2, even in the ideal case). The expected accuracy of these temperature readings can be estimated as follows. The achieved accuracy in time domain strongly depends on the bandwidth of the system and on the Signal-to-Noise Ratio (SNR). For an 80 MHz bandwidth, an accuracy of 0.3–0.6 ns is achievable. In our case, the achievable temperature accuracy would therefore be close to ~1–2 °C. In the worst case, this would still translate into a residual pressure uncertainty of ~2 mbar (~1.5 Torr), which is too large a value for practical applications.

Nevertheless, the obtained approximate temperature value would still provide essential information that could be refined using phase analysis, and additional information from one extra reflector. The extra reflector (Rph) shall be located between R1 and R2, and far enough from the edge of the membrane to stay unaffected by pressure. The propagation time between R1 and Rph shall be set to ~250 ns. In this configuration, a temperature variation from 0 °C to 50 °C would make the value of the differential phase ϕ_(Rph–R1)_ change by 6π. This would make it possible to achieve high temperature accuracy within the temperature operation range, as the phase of a given peak can itself be measured with high accuracy (±0.05–0.1 rad). With a total differential phase shift of 6π, the achievable temperature accuracy would be close to ±0.12–0.25 °C. However, this would generate phase ambiguity issues, as one phase value would correspond to three possible temperature values. The approximate temperature information obtained before would finally be used here, to resolve the ambiguity. Such a solution should eventually make it possible to achieve high accuracy temperature readings (better than ±0.25 °C), which would, in any case, be sufficient to compensate for the effect of the residual temperature sensitivity on pressure readings. It will be the purpose of future work to implement this solution.

### 4.2. Membrane Buckling

**Figure 8:**
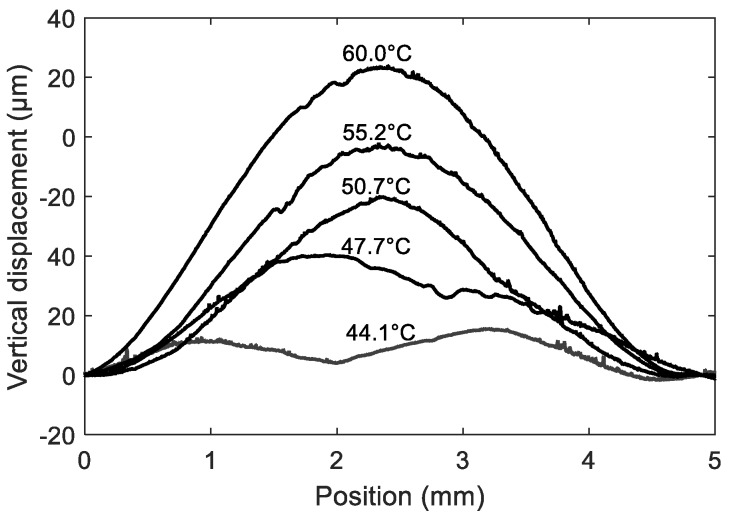
Evolution of the shape of the membrane versus temperature, in the absence of applied pressure (i.e., deformation due to temperature changes only). The measurements were done using a Dektak XT-A Surface Profilometer.

The thermally induced stress and strain fields can also result in a more problematic change in the device behavior, as they can lead to the buckling of the membrane above a given temperature threshold. We believe that buckling explains the discontinuity observed at ~43 °C, in the phase versus temperature curves of peaks R2, R3 and R4, obtained for the selected sensor (see Figure 7c). The buckling hypothesis is supported by preliminary profilometry measurements of the shape of the membrane versus temperature, performed at zero pressure (i.e., the pressure stayed constant and equal to 1 bar, on both sides of the membrane, during the whole experiment) (see Figure 8). The deformation clearly increases with temperature, and sudden shape changes can be observed, between 44 °C and 50 °C. The already deformed state, at ~44 °C is due to residual stress, which is generated in the LN/Si structure after wafer bonding, and concentrates in the membrane after the underlying Si is etched away. Interestingly, the shape becomes more regular at higher temperature, when the thermally induced deformation gets larger. Future work includes thorough characterization of the membrane shape at lower temperatures, in the range 20–44 °C.

Possible solutions to the buckling issue include thermal annealing (to reduce residual stress and shift the buckling point outside the temperature operating range of the sensor), and the pre-stressing of the membrane, to operate beyond the buckling point of the membrane, i.e., in a ‘buckling-free’ deformation range. A practical implementation of this solution would require the membrane to be equipped with a sealed vacuum cavity, deposited on top of the SAW track, and therefore located on the same side of the membrane as the IDT and reflectors. Vacuum would be preferred to any encapsulated reference pressure, as changes in temperature would also affect the reference pressure. The cavity should therefore be perfectly sealed, with no negative impact of the cavity walls on the propagating SAWs. Such a solution would certainly be costly, and difficult to implement.

**Figure 9:**
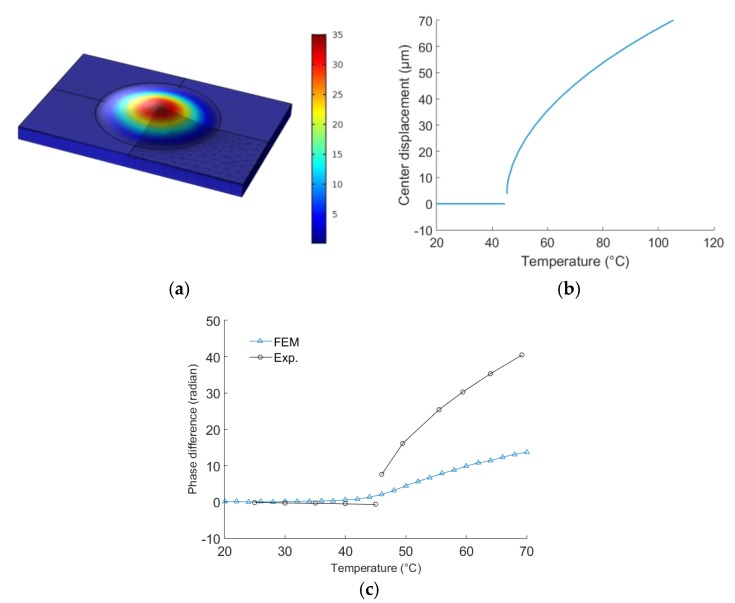
(**a**) Deformed shape of the membrane after buckling, at 60 °C. Only one fourth of the model was used to perform the computations, following the symmetry of the system; (**b**) Displacement vs. temperature, at the center of the membrane; (**c**) Sensor’s phase response vs. temperature, in the complete operating temperature range, including buckling effect.

It would therefore be better to solve the issue by changing or adjusting existing design parameters like, among others, the shape and thickness of the membrane. To do that, it is necessary to develop a simulation model first, which accounts for the observed buckling phenomenon. We built such a 3D model using Comsol Multiphysics. The model was used to perform thermo-mechanical studies of the membrane. The model takes advantage of the symmetries of the geometry, material and loads, which makes it possible to represent one fourth of the entire structure only. This simplification is acceptable, as the first buckling mode presents the same symmetry. Further simplification is achieved, by not taking the IDT, reflectors and Au/Cr bonding layer into account. In the model, the thickness of the membrane is exactly 30 μm. The boundary conditions include fixed displacements at the bottom of the substrate. The temperature is considered homogeneous, in the entire structure. No residual stress is considered, in the model. Therefore, the membrane is perfectly flat, at the initial temperature T0. An eigenvalue analysis made it possible to compute the buckling temperature as well as the deformed shape of the membrane, after buckling. The computations yielded a buckling temperature of 44.5 °C, which constitutes a bifurcation point. Above this point, the membrane can deform (buckle) in one of the two possible directions, up and down. As both directions are equivalent, a solution must be found to impose the deformation in one chosen direction, numerically. This was achieved by fixing a level of deformation for the membrane (i.e., by applying a pressure or a force on it), before letting the FEM software look for the temperature that would generate this pre-defined deformation, in the buckled state (see Figure 9a,b). The results show a maximal displacement of 35 μm at 60 °C. This is 40% larger than the experimentally observed displacement (see Figure 8). The stress and strain fields along the acoustic path of the sensor were also computed. Therefore, it became possible to compute the additional (unwanted) sensor sensitivity to temperature in the buckled state, following the method described in Reference [24]. The resulting temperature sensitivity (after buckling) in phase domain is shown in Figure 9c. It is also compared to the measured ‘phase vs. temperature’ characteristic curve. The model overestimates the temperature sensitivity after buckling, but it gives the right tendency. Therefore, it is already possible to use it for design improvement purposes. Future work will include the testing of different membrane shapes (including elliptical membranes), to shift the buckling point beyond 45 °C.

## 5. Conclusions

We presented a solution to measure pressure, using a SAW R-DL pressure sensor. The sensor is based on an innovative LN/Si structure. The 2.45 GHz R-DL fabrication problems were solved using a combination of e-beam and optical lithography. The inconvenient capacitive coupling, which arises between the two connection pads of the Inter Digital Transducer due to the presence of the Au/Cr bonding layer, was strongly reduced using much smaller pads. This also helped reduce the amount of energy lost through BAW emission in Si. The use of an extra, viscous damping layer on the back side of the chip also helped reduce the amplitude of the spurious BAW peaks. Besides, it was shown that thermo-mechanical effects are responsible for the observed residual temperature sensitivity. Solutions to get rid of this issue include the use of one additional reflector, to actively measure temperature, in addition to pressure. Thermo-mechanical strain and stress fields were also shown to be responsible for the discontinuities observed in the response curves of the sensor. They are due to the buckling of the membrane, at a threshold temperature. Possible solutions were proposed and discussed.
